# Community-based Health Workers Achieve High Coverage in Neonatal Intervention Trials: A Case Study from Sylhet, Bangladesh

**DOI:** 10.3329/jhpn.v28i6.6610

**Published:** 2010-12

**Authors:** Rasheduzzaman Shah, Melinda K. Munos, Peter J. Winch, Luke C. Mullany, Ishtiaq Mannan, Syed Moshfiqur Rahman, Radwanur Rahman, Daniel Hossain, Shams El Arifeen, Abdullah H. Baqui

**Affiliations:** ^1^ ICDDR,B, Mohakhali, Dhaka 1212, Bangladesh; ^2^ Department of International Health, Johns Hopkins Bloomberg School of Public Health, Baltimore, MD 21205-2103, USA; ^3^ Save the Children-USA, Bangladesh Country Office, Dhaka, Bangladesh

**Keywords:** Chlorhexidine, Cluster-randomized trial, Community-based studies, Community health workers, Interventions, Neonatal health, Umbilical cord cleansing, Bangladesh

## Abstract

A large proportion of four million neonatal deaths occur each year during the first 24 hours of life. Research is particularly needed to determine the efficacy of interventions during the first 24 hours. Large cadres of community-based workers are required in newborn-care research both to deliver these interventions in a standardized manner in the home and to measure the outcomes of the study. In a large-scale community-based efficacy trial of chlorhexidine for cleansing the cord in north-eastern rural Bangladesh, a two-tiered system of community-based workers was established to deliver a package of essential maternal and newborn-care interventions and one of three umbilical cord-care regimens. At any given time, the trial employed approximately 133 community health workers—each responsible for 4–5 village health workers and a population of approximately 4,000. Over the entire trial period, 29,760 neonates were enrolled, and 87% of them received the intervention (their assigned cord-care regimen) within 24 hours of birth. Approaches to recruitment, training, and supervision in the study are described. Key lessons included the importance of supportive processes for community-based workers, including a strong training and field supervisory system, community acceptance of the study, consideration of the setting, study objectives, and human resources available.

## INTRODUCTION

An estimated four million children die each year during the first 28 days of life (1, 2), a large proportion of which occur during the first 24 hours. Recent studies have documented that 41% and 32% of neonatal deaths occur during the first day in rural Ghana (3) and rural India respectively (4). A number of interventions with demonstrated or potential efficacy in decreasing neonatal mortality can be delivered during the first 24 hours of life and include early initiation of breastfeeding, dosing of vitamin A to newborns, and care for asphyxiated newborns. These and other interventions are commonly included in packages of newborn-care interventions (5–7).

There is an important research agendum relating to newborn-care interventions during the first day of life. The agendum includes determining the efficacy of different technical interventions to be delivered during this time and identifying solutions to operational constraints to delivery of the interventions in the right way at the right time. Community-based trials of newborn-care interventions face challenges in ensuring prompt notification of new births and provision of interventions to newborns within the first day of life. Moreover, neonatal deaths, even in settings with high mortality, are relatively rare, and trials aiming at showing a mortality effect for these interventions often require large sample-sizes. As a result, large cadres of community-based health workers have been used for delivering these interventions, presenting operational challenges for the implementation of trials, including recruitment and training of staff responsible for the delivery of interventions and collection of data, and decisions about how to optimize the delivery of interventions.

Cleansing of the umbilical cord with chlorhexidine is a prime example of a technical intervention whose efficacy depends on application within the first day of life. Topical applications of chlorhexidine to the umbilical cord-stump of newborns may reduce omphalitis and systemic infections originating from exposure of the cord-stump (8). Application of chlorhexidine immediately after birth may both prevent passage of microbes through the patent umbilical stump immediately after birth and also prevent infection of the umbilical stump and surrounding skin (9). In a trial of chlorhexidine for cleansing the umbilical cord in a rural community in Nepal where more than 90% of babies are born at home, mortality was reduced by 24% among infants receiving chlorhexidine compared to those who received no application in the cord-stump [‘dry cord-care’, the present recommendation of the World Health Organization (WHO)] (10). About two-thirds of infants were reached within 24 hours of birth, and a protective effect of cleansing the umbilical cord with chlorhexidine among this subset was evident. Severe infection was reduced by 87% and mortality by 34% in the chlorhexidine group compared to dry cord-care among those enrolled within 24 hours while no difference was observed between the groups among those enrolled after 24 hours.

This paper presents a case study of the practical implications and operational challenges associated with the deployment of large cadres of community-based workers to evaluate neonatal interventions: the two-tier system of community-based health workers established as part of a large, cluster-randomized efficacy trial of chlorhexidine for cleansing the umbilical cord in rural Bangladesh (11). We describe the role of workers in this system used for delivering the chlorhexidine intervention and collection of data; discuss the practical aspects and challenges of implementing the system, including recruitment, training, and monitoring; and present the coverage levels achieved. While this represents a much more intensive and costly system than would be appropriate for implementation under routine programmatic conditions, there are lessons learnt from this study that are applicable to programmes.

## MATERIALS AND METHODS

### Study design and intervention content

The second phase of the Project for Advancing the Health of Newborns and Mothers study (Projahnmo II) in rural areas of Sylhet district, Bangladesh, is a cluster-randomized efficacy trial of 4.0% chlorhexidine for cleansing the umbilical cord in neonates. The objectives of this trial included validating the findings of the previous trial in Nepal and expanding the evidence base by additionally assessing the efficacy of a single cleansing compared to dry cord-care. The first phase of Projahnmo, described elsewhere (12), employed female community health workers (CHWs) to deliver a package of essential care for the newborns; these CHWs provided the foundation for the cadre system of community-based workers established for Projahnmo II (11).

The study population for this community-based trial included all liveborn infants delivered in 22 unions (administrative units) in three rural subdistricts (Zakiganj, Kanaighat, and Beanibazar) of Sylhet district. The population in these subdistricts totalled approximately 545,000 at the start of the study, with 12,000–13,000 births per year. The study area was divided into 133 CHW clusters, each of which was randomly allocated to one of three cord-care regimens. Enrolled newborns in each cluster received the cord-care regimen assigned to the cluster in which they were born. The three cord-care regimens were: (a) cleansing of the umbilical cord with 4.0% chlorhexidine as soon as possible after birth and on subsequent days until the age of seven days; (b) a single cord cleansing with 4.0% chlorhexidine as soon as possible after birth; and (c) the WHO-recommended cord-care intervention (dry cord-care), which was treated as the comparison group. Dry cord-care, as defined by the WHO, involves handwashing with clean water and soap before and after care of the cord; keeping the cord dry and exposed to air or loosely covered with a clean cloth; cleansing the cord when necessary with clean water and soap; and avoiding the application of unclean substances or bandages, or unnecessary touching of the cord (10). The dry cord-care intervention delivered in Projahnmo II was based on the WHO's recommendation of clean and hygienic practices surrounding the tying and cutting of the cord and the avoidance of topical applications to the cord. In addition, enrolled mothers and newborns in all the three study arms received a community-based package of essential newborn care similar to the one provided in the previous study in this area (11, 12).

### Cadre system of community-based workers

**Fig. 1.**
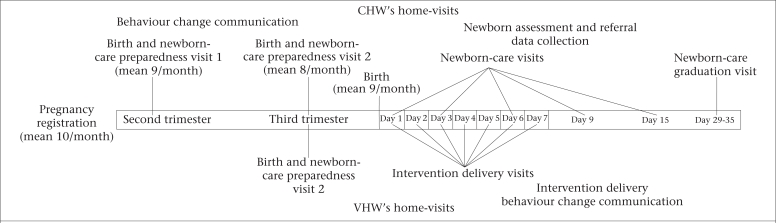
Division of responsibility between CHWs and VHWs in cluster-randomized trial of chlorhexidine for cleansing the umbilical cord

To deliver the chlorhexidine intervention in a standardized manner, two cadres of female health workers—CHWs and village health workers (VHWs)—were recruited and trained. In this study, CHW referred to a higher-level community-based worker with some supervisory and quality-assurance responsibilities whereas VHWs were lower-level workers with a narrow range of tasks. Figure 1 presents the division of responsibilities between the two cadres.

CHWs=Community health workers;
VHWs=Village health workers

VHWs were the frontline workers through whom the chlorhexidine intervention was provided to newborns. They were generally older local women (mean age 33 years, range 15–80 years), often married or widowed (81%). Many had no formal education when recruited (49%), and 45% were traditional birth attendants (TBAs). Each VHW was responsible for approximately 200 households (equivalent, on average, to approximately 2 enrolled newborns per month, or 3 newborn-care visits per week), and their main task was to provide umbilical cord-care during home-visits, including application of chlorhexidine in the two intervention arms and delivery of specific newborn-care messages in all the three arms. Their participation in data-collection activities was limited to recording the time and date of daily visits after birth and whether they had delivered the allocated intervention.

VHW was an effective informant in birth and pregnancy surveillance activities in her community. As local residents, VHWs were well-informed about new births and pregnancies within the catchment area with a smaller population-size. In the case of VHWs who had been working as TBAs before starting their work for the study, their pre-study roles in maternal and newborn-care also helped them in their birth and pregnancy-surveillance tasks.

CHWs were responsible for more complex tasks, requiring higher levels of education and training compared to VHWs. Their surveillance responsibilities included maintenance of a register of married women of reproductive age and identification and tracking of pregnant women through fortnightly visits to all households in their catchment area. Other responsibilities included provision of behaviour change communication material during home-visits; assessment of newborns for basic signs of morbidity; data collection; supervision of, and providing support to, VHWs; and quality assurance for VHW activities. Each CHW was responsible for 4–5 VHWs and a population of approximately 4,000 (mean 4,109, range 2,017–5,598). At the time of recruitment, CHWs were generally young (mean age 25 years, range 17–51 years) unmarried women (64%) with at least a 10^th^ grade education.

Pregnancy surveillance was a scheduled task for CHWs, and each CHW made a visit to every household fortnightly. VHWs were recruited from the local community and assigned for a population of 1,000 (approximately 180–200 households). Given the poor literacy rate among VHWs, they were not given any paperwork to record pregnancies within their assigned community. Rather, during their personal contacts and communication within the community, when VHWs came to know about a new pregnancy, they informed respective CHWs either by mobile phone or by meeting in person during the weekly field-based feedback session. And thereafter, registration and enrollment of reported new pregnancy was assigned to the respective CHWs.

The unit of randomization for this study was the geographical cluster; each cluster was assigned to a single CHW. Thus, all VHWs, supervised by a particular CHW, delivered the same intervention. Randomization of the smaller VHW areas was initially considered because it would have been more statistically efficient than randomization of CHW areas. However, randomization by VHW area would have required each CHW to supervise the delivery of all three interventions. Randomization by VHW area would also have presented an increased risk of contamination compared to randomization by CHW area because VHW clusters were small.

## RESULTS

### Implementation of the CHW-VHW system

#### Recruitment

Following circulation of a job advertisement, representatives from the partner implementing organizations interviewed applicants and selected the contingent of CHWs on successful completion of training. We recruited local women with a 10th grade education. While we aimed at hiring women from the clusters in which they would work, we did not receive sufficient applications from eligible local women in some clusters. In eight clusters, we, therefore, hired CHWs from outside the study area or assigned CHWs to clusters that were different from their areas of residence. We organized community-level sensitization workshops in areas not included in the previous study, disseminated prior study results, and informed community opinion leaders and residents about the work performed by CHWs and VHWs.

#### Training

**Fig. 2.**
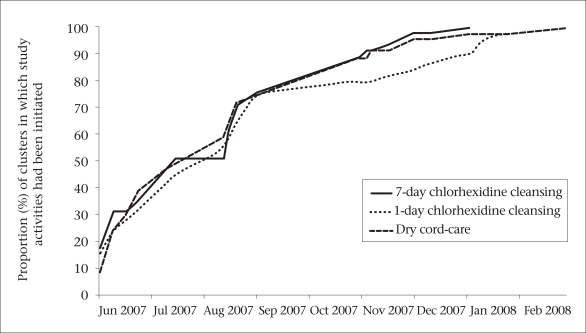
Timing of intervention phase-in in three study arms of cluster-randomized trial of chlorhexidine for cleansing the umbilical cord

The randomization scheme and logistical concerns influenced the organization and timing of training and phase-in of intervention implementation. CHWs and VHWs from each arm were trained separately to avoid receiving conflicting information relating to the content of the different intervention arms and to ensure that training messages were relevant to the activities assigned to each worker. At the same time, instruction across groups of CHWs was standardized to ensure that training content differed only with respect to the cord-care intervention to which the cluster was allocated. Due to limitation of space, personnel, and funding, workers were trained in batches of approximately 20 over a six-month period, resulting in a gradual phase-in of study activities. As batches of CHWs and VHWs completed their training, the study activities were initiated in those clusters. To ensure approximate balance across the study arms during the phase-in period, the timing of selection and training of workers were evenly distributed over the three arms (Fig. 2).

Training of CHWs covered behaviour change communication, assessment of newborns (including recognition of signs of illness and interview of caregiver regarding the health status of the baby), data collection, and supervision over a period of 67 days, 25 of which were spent in the field. In addition to classroom instruction, CHWs practised counselling the pregnant mothers, assessing the newborns, and identifying and classifying the sick newborns at the delivery ward of the Sylhet Osmani Medical College Hospital and updated a register of married women of reproductive age in their assigned clusters to familiarize themselves with the management information system.

Following the training of CHWs, each CHW conducted a three-day training session for 4–5 VHWs assigned to her under the supervision of a field supervisor. In total, 30 field supervisors (each supervised 4–5 CHWs) were engaged in the study. One-third of the supervisors were promoted who worked as CHWs during the previous phase of the study, considering their performance as CHWs in previous phase and their previous experience. The other 20 CHWs were recruited following standard administrative procedures. Successful completion of 12th grade education was the mandatory benchmark for hiring field supervisors. A team comprising 3 field research officers, 2 field managers, and 2 trainers facilitated all the training sessions for field supervisors. Later, the field supervisors joined this training team to facilitate the training of CHWs. Hiring of VHWs was contingent upon the successful completion of training. The educational qualifications of VHWs, however, were less demanding than of CHWs, and the identification of eligible candidates focused on community acceptance and an expressed willingness to work for Projahnmo.

The training covered the timing of household-visits and messages to be delivered during the visits, cord-cleansing procedure (in the intervention arms), simple record-keeping (Appendix 1), and planned contacts between the CHW and the VHW. Since many VHWs were illiterate, the training and memory-aids were primarily visual and pictorial (samples shown in Appendix 2). Training on the cord-cleansing procedure included a demonstration followed by practice using dolls. The VHWs were given pictorial instructions for the cleansing cord to help standardize application, and the data-collection forms included simple pictograms (Appendix 1). During supervisory visits in the phase-in period, some VHWs were observed applying chlorhexidine in a manner slightly different from the instructed method. To improve performance, a video demonstrating the correct application of chlorhexidine was developed and shown to the VHWs on laptops.

#### Supervision, monitoring, and feedback system

A tiered supervision system was implemented, in which the CHWs supervised the VHWs, and the field supervisors supervised the CHWs. The approach to supervision emphasized field-based observation and elements critical to the quality of the intervention, such as ensuring that chlorhexidine was applied according to the standardized method. Each field supervisor was responsible for 5–6 CHWs and visited each CHW 2–3 times per fortnight. The supervisors used a field observation checklist to record their findings and observations and conducted quality checks of completed data-collection forms. These findings were shared in fortnightly review meetings with the CHWs and used for identifying the low-performing CHW whose skills were strengthened through targeted re-orientation sessions and on-the-job refresher training. The field supervisors also provided immediate feedback and counselling to the CHWs and VHWs if they observed weaknesses in the delivery of interventions or counselling. Many CHWs reported problems during the initial months of the study in providing guidance to the VHWs and using the supervisory checklist effectively. The field supervisors, therefore, provided one-on-one refresher training to the CHWs with weaker supervisory skills.

Accurate identification of signs of umbilical cord infection was critical to the internal validity of the study. Given the subjective nature of the signs, there are considerable challenges involved in training community-based workers to recognize these signs consistently and classify their severity. Previous community-based work in Nepal (11, 13) and Tanzania (14) has used a standardized set of 50 digital images of umbilical cords with varying degrees of inflammation to assess intra- and inter-worker agreement in recognition of signs and to estimate sensitivity and specificity of workers compared to a physician gold standard ranking of the images (13). Following this model, the CHWs were trained to recognize and classify the severity of three signs of infection, such as redness, pus, and swelling. To evaluate their skills, they were asked to assess the images of umbilical cords for signs of infection. This assessment was repeated quarterly, and the results were used for identifying those CHWs needing more focused one-on-one training. A formal analysis of the intra- and inter-rater agreement indicators for the CHWs is pending.

Data collected in the field were tallied, and basic analysis on selected monitoring indicators was done manually by the CHWs and their supervisors at the field office during the fortnightly review meetings. The CHWs also relayed lessons from real-time data reviews to the VHWs assigned to them during their routine field-based meetings. This built-in system of real-time data processing enabled the study managers to track key indicators, such as pregnancy-surveillance rates, birth rates, newborn-care visits conducted, and, most critically, timing and coverage of intervention delivery and to quickly identify and address problems.

### Coverage

**Fig. 3.**
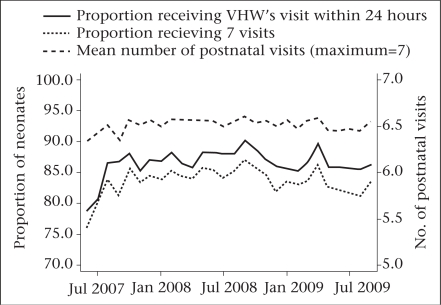
Coverage of postnatal visits by VHWs to deliver umbilical-care interventions

There were 35,908 babies born alive during the trial period of June 2007–September 2009. Of them, 29,760 (83%) were met alive by the VHWs within seven days and enrolled in the study. Figure 3 presents trends in the coverage of home-visits after birth to deliver behaviour-change messages and the allocated cord-care regimen. Of the 29,760 enrolled neonates, 25,858 (87%) received a visit by the VHWs within 24 hours of birth.

Challenges to achieving high levels of coverage included delays in receiving notification of new births and the time required to travel to households with a birth. To facilitate notification of new births, the VHWs and CHWs used mobile phones to communicate among themselves and with the families of pregnant women. Because of the size of the area for which they were responsible, the CHWs were given travel allowances, and they used public transport while the VHWs generally visited households on foot. Flooding during the monsoon made travel difficult at times; in September 2007, most fieldwork was halted for four weeks due to floods. While the VHWs still attempted to visit neonates to deliver the intervention, antenatal visits by the CHWs were largely halted during this time.

## DISCUSSION

Our experience in Projahnmo-II demonstrates the feasibility of establishing and employing large cadres of community-based workers to deliver a newborn-care intervention in a standardized manner, on a large scale, and with high coverage in a low-resource setting. While many issues described may be common to other intervention trials, our intention was not to suggest a model for neonatal intervention trials or programmes but rather to provide a case study of a system for the delivery of intervention and monitoring for a large-scale community-based efficacy trial of a neonatal health intervention. We discuss here some strategies we employed with potential relevance to the design and establishment of community-based cadres in other large-scale programmes and studies of neonatal interventions, particularly in low-resource settings.

Our two-tier system of workers matched the resources of the study area to the needs of the study. Since this trial aimed at estimating the efficacy of chlorhexidine for cleansing the cord on neonatal mortality and infection of the cord, timely and correct application of chlorhexidine in the intervention arms was critical. Given prior evidence that the impact of chlorhexidine is greater when it is applied within 24 hours of birth, we emphasized initiation of the chlorhexidine intervention within this time period. As for other newborn interventions delivered in the first 24 hours of life, a key challenge was to ensure that the CHWs received timely notification of births and were then able to reach the household quickly in an area where transport can be difficult. To address this challenge, we established a large cadre of less-skilled VHWs, trained to deliver the intervention, who had small catchment areas and strong connections with the communities within which they worked by virtue of their local residency and, for some, their status as TBAs. Since the literacy and educational levels among the study women, including the VHWs, were low, we relied on a more limited number of CHWs, with their higher levels of education and more extensive training, to perform more complex data collection and assessments of newborns needed for the trial and to supervise the VHWs. This approach, with two complementary cadres of community-based workers, may be useful in other settings where large numbers of newborns must be reached soon after birth and/or multiple tasks requiring distinct types of expertise, or training must be completed during home-visits. In any setting, a decision on the optimal deployment of community-based worker cadres would include an assessment of costs incurred under this model and a range of possible alternatives. Further, the purpose of the deployment, i.e. research vs surveillance vs programmatic delivery of services, the context-specific needs, and the resources available will play an important role in decision-making.

An important aspect of the approach we used is that the CHWs and VHWs in our study were paid and supported through extensive training, monitoring and supervision activities that required substantial human and financial resources. This level of support, while appropriate for an intervention trial, is unrealistic under routine programmatic conditions. However, attention to the mechanisms for support of community-based health workers (training, supervision, and payment) may still be instructive for programme managers, even if these are implemented in a less resource-intensive manner. Results of reviews of programmes that employed community-based health workers suggest that the support provided for the workers is an important determinant of programmatic success (15, 16), and the same may be true for intervention trials. The staff members interviewed for this study cited several other factors as key to the successful establishment of the cadres and provision of the intervention, including (a) highly motivated staff, most of whom, at the field level, were local; (b) frequent, two-way communication between the main office and the field; (c) a strong supervisory system and the ability to provide rapid feedback to VHWs and CHWs; and (4) some degree of baseline acceptance by the community and advocacy to increase acceptance by the community.

In countries with the highest rates of neonatal mortality, most deliveries take place typically at home, and families may be reluctant to leave the home during the first month of life for various social and cultural reasons (17). To achieve high coverage under these conditions in community-based neonatal-care intervention trials such as this one, cord-cleansing material like chlorhexidine can be delivered in the home by trained TBAs, CHWs working in collaboration with TBAs, or CHWs working alone (12, 18–20).

Routine programmes may seek to have family members or TBAs apply chlorhexidine for cord cleansing at deliveries in the home, with the role of CHWs or community-level cadre of workers focussed on raising awareness about newborn's health and instruction to caregivers on how to apply chlorhexidine correctly. Under such circumstances, CHWs would be recruited and trained in a very different manner. The focus of training might be how to promote chlorhexidine and other maternal and newborn-care practices in an integrated manner, how to demonstrate correct application on a doll to family members and TBAs, and how to address concerns community members might have about chlorhexidine. These research questions were addressed in a small-scale operational research study implemented in an adjacent area at the time of the cluster-randomized trial discussed in this paper. A parallel effort might be necessary to institute application of chlorhexidine for cord cleansing in facility-based deliveries, where problems with newborn infections again are important.

## ACKNOWLEDGEMENTS

Funding for the PROJAHNMO Phase 2 Trial is provided by the United States Agency for International Development, Office of Health, Infectious Diseases, and Nutrition, Global Health Bureau and the Dhaka Mission through the Global Research Activity Cooperative Agreement (No. GHS-A-00-03-00019-00), and the Saving Newborn Lives initiative of Save the Children Federation-USA through a grant from the Bill & Melinda Gates Foundation.

The authors thank the study participants and field staff, particularly CHWs and VHWs, for their efforts in implementing the study and delivering the intervention, and the Ministry of Health and Family Welfare, Government of Bangladesh, for their support and collaboration in all phases of the study.
